# Insecticidal Activity of Essential Oil of *Carum Carvi* Fruits from China and Its Main Components against Two Grain Storage Insects

**DOI:** 10.3390/molecules15129391

**Published:** 2010-12-20

**Authors:** Rui Fang, Cai Hong Jiang, Xiu Yi Wang, Hai Ming Zhang, Zhi Long Liu, Ligang Zhou, Shu Shan Du, Zhi Wei Deng

**Affiliations:** 1 Protection and Utilization of Traditional Chinese Medicine of Beijing Area Major Laboratory, Beijing Normal University, Haidian District, Beijing 100875, China; 2 Department of Entomology, China Agricultural University, Haidian District, Beijing 100094, China; 3 Analytical and Testing Center, Beijing Normal University, Haidian District, Beijing 100875, China; 4 Department of Plant Pathology, China Agricultural University, Haidian District, Beijing 100094, China

**Keywords:** *Carum carvi*, *Sitophilus zeamais*, *Tribolium castaneum*, fumigant, contact toxicity, essential oil, (*R*)-carvone, D-limonene

## Abstract

During our screening program for agrochemicals from Chinese medicinal herbs and wild plants, the essential oil of *Carum carvi* fruits was found to possess strong contact toxicity against *Sitophilus zeamais* and *Tribolium castaneum* adults, with LD_50_ values of 3.07 and 3.29 μg/adult, respectively, and also showed strong fumigant toxicity against the two grain storage insects with LC_50_ values of 3.37 and 2.53 mg/L, respectively. The essential oil obtained by hydrodistillation was investigated by GC and GC-MS. The main components of the essential oil were identified to be (*R*)-carvone (37.98%) and D-limonene (26.55%) followed by α-pinene (5.21), *cis*-carveol (5.01%) and β-myrcene (4.67%). (*R*)-Carvone and D-limonene were separated and purified by silica gel column chromatography and preparative thin layer chromatography, and further identified by means of physicochemical and spectrometric analysis. (*R*)-Carvone and D-limonene showed strong contact toxicity against *S. zeamais* (LD_50_ = 2.79 and 29.86 μg/adult) and *T. castaneum* (LD_50_ = 2.64 and 20.14 μg/adult). (*R*)-Carvone and D-limonene also possessed strong fumigant toxicity against *S. zeamais* (LC_50_= 2.76 and 48.18 mg/L) and *T. castaneum* adults (LC_50_= 1.96 and 19.10 mg/L).

## 1. Introduction

During our screening program for new agrochemicals from local wild plants and Chinese medicinal herbs, the essential oil from *Carum carvi* fruits (Family: Apiaceae) was found to possess insecticidal activity towards the maize weevil, *Sitophilus zeamais* (Motsch) and red flour beetle, *Tribolium castaneum* Herbst. Common caraway (*C. carvi*), one of the oldest herbs known and with a pleasant aroma, is native to Asia, Europe, and North Africa. Its fruits are used in pharmacy, perfumery and food. The dried ripe fruits of *C. carvi* are used in traditional Chinese medicine and other folk medicines as a carminative, since it is effective against spasmodic gastrointestinal complaints, flatulence, irritable stomach, indigestion, lack of appetite, and dyspepsia in adults [1]. The chemical composition of the essential oil of *C. carvi* collected from various countries has been widely studied [2,3,4,5,6] and great variations in essential oil content and chemical composition of the essential oil were observed. Many data indicated the essential oil possessed antimicrobial, antifungal, molluscidal, nematicidal, antioxidant and antiaflatoxigenic activities, as well as potential as a cancer preventing agent [3,6,7,8,9,10,11]. The essential oil of *C. carvi* has been demonstrated to possess strong contact and fumigant toxicity as well as repellency against several insects and mites, e.g. Japanese termite (*Reticulitermes speratus*) [12], rice weevil (*S. oryzae*) [13], sciarid fly *Lycoriella ingenua* larvae [14], the two-spotted spider mite *Tetranychus urticae* and its predator *Phytoseiulus persimilis* [15], and the poultry red mite *Dermanyssus gallinae* [16]. However, no reports on insecticidal activity of essential oil of *C. carvi* fruits collected from China against stored product insects exist. In this paper, we report the contact and fumigant toxicities of the crude essential oil and two main components derived from the essential oil against two species of grain storage insects.

## 2. Results and Discussion

### 2.1. Chemical Composition of the Essential Oils

The chemical composition of the essential oil derived from *C. carvi* fruits collected from China was shown in Table 1. The main constituents of *C. carvi* essential oil were (*R*)-carvone (37.98%) andD-limonene (26.55%) followed by α-pinene (5.21), *cis*-carveol (5.01%) and β-myrcene (4.67%). A total of 30 components were identified in the oil, accounting for 97.58% of the total oil (Table 1).

The results were different from previous reports. For example, an essential oil of *C. carvi* collected in Qinghai, China contained (*R*)-carvone (51.62%) and D-limonene (38.26%) as its two main components [17]. (*R*)-carvone and D-limonene were also found to be the two main constituents in the essential oils collected from Europe and North America [2,5,6,7,8]. However, according to some reports these two compounds were not detected in the essential oil of *C. carvi*. For example, caraway essential oil cultivated in Iran contained γ-terpinene (24.40%), 2-methyl-3-phenylpropanal (13.20%) and 2,4(10)-thujadiene (14.02%) as its main constituents [24] while in another report on the essential oil of *C. carvi* from Iran [3] cuminaldehyde (22.08%) and γ-terpinene (17.86%) were the two main constituents, followed by *p*-cymene (7.99%). The main components of the essential oil of *C. carvi* seeds from Bangladesh were thymol (48.20%), *o*-cymene (19.29%), γ-terpinene (17.61%) and trimethylene dichloride (8.81%) [23]. The above findings suggest that further studies on plant cultivation and essential oil standardization are needed because the chemical composition of the essential oil varies greatly with the plant population.

**Table 1:** Chemical composition of the essential oil of *Carum carvi* fruits.

Compounds	RI *	Relative content (%)
α-Pinene	939	5.17
β-Pinene	974	0.02
β-Myrcene	991	4.67
*p*-Cymene	1026	0.34
D-Limonene	1029	26.55
1,8-Cineol	1032	0.45
Linalool	1097	0.87
*cis*-Limonene oxide	1132	0.63
Menthone	1152	0.59
Menthol	1172	0.07
4-Terpineol	1174	1.34
γ-Terpinene	1179	0.17
*p*-Cymen-8-ol	1182	0.49
Dihydrocarvone	1200	1.21
*cis*-Carveol	1226	5.01
Cuminic aldehyde	1236	0.06
(*R*)-Carvone	1242	37.98
Dihydrocarveol	1253	1.08
α-Terpinene-7-al	1277	1.04
Bornyl acetate	1285	0.94
Cumin alcohol	1288	0.72
Eugenol	1356	1.45
Methyleugenol	1401	0.93
Germacrene D	1479	2.81
Myristicin	1513	0.05
Elemicine	1554	0.19
Caryophyllene oxide	1583	1.23
Apiol	1682	0.41
Phytol	2119	0.06
1,2-Benzenedicarboxylic acid, mono(2-ethylhexyl)ester	2549	1.05
Total		97.58

* RI, retention index as determined on a HP-5MS column using the homologous series of *n*-hydrocarbons as reference.

(*R*)-Carvone and D-limonene were further separated and purified by silica gel column chromatography and preparative thin layer chromatography based on bioassay-guided fractionation and were characterized from their ^1^H-, ^13^C-NMR and mass spectra. After comparing the physicochemical and spectrometric data with those reported in the literature [20,21,22], the identities of the two compounds were further confirmed. 

### 2.2. Insecticidal Activity

The essential oil of *C. carvi* showed strong contact toxicity against *S. zeamais* and *T. castaneum* adults with LD_50_ values of 3.07 and 3.39 μg/adult, respectively (Table 2). Compared with the famous botanical insecticide, pyrethrum extract (25% pyrethrine I and pyrethrine II), the essential oil was nine times less active against *T. castaneum* adults because pyrethrum extract displayed a LD_50_ value of 0.36 μg/adult (Table 2). However, it exhibited the same contact toxicity against *S. zeamais* (pyrethrum extract, LD_50_ = 4.29 μg/adult). 

**Table 3:** Contact toxicity of essential oil of *Carum carvi* and its main components (*R*)-carvoneand D-limonene against *Sitophilus zeamais* (SZ) and *Tribolium castaneum* (TC) adults.

Insect	Compounds	LD_50_* (95% FL)	LD_90_* (95% FL)	Slope ± SE	Chi square (χ^2^ )	df
SZ	Essential oil	3.07	4.34	8.52 ± 1.02	9.36	24
		(2.89-3.29)	(3.91-4.67)			
	(R)-Carvone	2.79	3.93	8.66 ± 0.94	7.44	24
		(2.53-3.06)	(3.67-4.05)			
	D-Limonene	29.86	44.86	7.38 ± 0.84	8.64	24
		(27.28-30.10)	(41.49-47.23)			
	Pyrethrum extract**	4.29 (3.86-4.72)	-	-	-	-
TC	Essential oil	3.39	6.08	5.06 ± 0.63	12.48	24
		(3.11-3.72)	(5.57-6.54)			
	(R)-Carvone	2.64	4.27	6.14 ± 0.88	6.27	24
		(2.41-2.93)	(3.91-4.56)			
	D-Limonene	20.14	43.58	1.59 ± 0.22	16.02	24
		(18.45-21.89)	(39.85-46.91)			
	Pyrethrum extract**	0.36 (0.32-0.41)	-	-	-	-

* Concentration (μg/adult) ** data from Liu *et al.* [23].

The essential oil of *C. carvi* fruits also possessed strong fumigant activity against *S. zeamais* and *T. castaneum* adults with LC_50_ values of 3.37 and 2.53 mg/L, respectively (Table 3). The currently used grain fumigant, methyl bromide (MeBr) was reported to have fumigant activity against *S. zeamais* and *T. castaneum* adults with LC_50_ values of 0.67 and 1.75 mg/L, respectively, thus the essential oil was five times less toxic to *S. zeamais* adults compared with the commercial fumigant MeBr. However, the essential oil exhibited the same fumigant toxicity to *T. castaneum* adults as MeBr. Compared with the other essential oils in the lieterature, the essential oil of *C. carvi* possessed stronger fumigant toxicity against *T. castaneum* adults, e.g. essential oils of *Murraya exotica* (LC_50_ = 6.84 mg/L) [38], *Mentha microphylla* (LC_50_ = 4.51 μL/L), *Citrus reticulata* (LC_50_ = 19.47 μL/L), *Schinus terebenthifolius* (LC_50_ = 20.50 μL/L) [39], *Perovskia abrotanoides* (LC_50_ = 11.39 μL/L) [40] and *Drimys winteri* (LC_50_ = 9.0-10.5 μL/L) [41], but lesser toxic than the essential oil of *Laurelia sempervirens* (LC_50_ = 1.6-1.7 μL/L) [41]. However, compared with the other essential oils in the previous studies, the essential oil of *C. carvi* exhibited stronger fumigant toxicity against the maize weevils, e.g. essentail oils of *M. exotica* (LC_50_ = 8.29 mg/L) [38], *Artemisia lavandulaefolia* (LC_50_ = 11.2 mg/L) [24], *A. vestita* (LC_50_ = 13.42 mg/L) [42], *Illicium simonsii* (LC_50_ = 14.95 mg/L) [43] and * A.* *sieversiana* (LC_50_ = 15.0 mg/L). The above findings suggest that the essential oil of *C. carvi* fruits show potential for development as a novel natural fumigant for stored products.

**Table 2:** Fumigant toxicity of essential oil of *Carum carvi* and its main components (*R*)-carvoneand D-limonene against *Sitophilus zeamais* (SZ) and *Tribolium castaneum* (TC) adults.

Insect		Compounds		LC_50_* (95% FL)		LC_90_* (95% FL)		Slope ± SE		Chi square (χ^2^)		df	
SZ		Essential oil		3.37		5.04		7.34 ± 0.84		6.48		24	
				(3.02-3.73)		(4.69-5.43)							
		(*R*)-Carvone		2.76		4.56		5.89 ± 0.56		14.40		24	
				(2.59-2.92)		(4.23-4.89)							
		D-Limonene		48.18		104.24		3.74 ± 0.40		14.88		24	
				(45.29-51.04)		(96.46-108.95)							
		MeBr**		0.67		-		-		-		-	
TC		Essential oil		2.53		5.47		3.83 ± 0.43		8.40		24	
				(2.38-2.68)		(4.97-5.89)							
		(*R*)-Carvone		1.96		3.67		4.65 ± 0.59		6.96		24	
				(1.83-2.12)		(3.41-3.93)							
		D-Limonene		19.10		29.19		7.25 ± 0.89		15.84		24	
				(17.53-20.04)		(27.22-31.57)							
		MeBr**		1.75		-				-		-	

* Concentration (mg/L air) ** data from Liu and Ho [37].

The isolated compound, (*R*)-carvone showed stronger contact toxicity against *S. zeamais* and *T. castaneum* adults (LD_50_= 2.79 and 2.64 μg /adult, respectively) than the other isolated compound, D-limonene (LD_50_= 29.86 and 20.14 μg /adult, respectively) (Table 2). Moreover, (*R*)-carvone also possessed stronger fumigant toxicity against *S. zeamais* and *T. castaneum* adults (LC_50_ = 2.76 and1.96 mg/L air, respectively) than D-limonene (LD_50_ = 48.18 and 19.10 mg/L air, respectively) (Table 3). Compared with the commercial fumigant MeBr, (*R*)-carvone exhibited the same fumigant toxicity against *T. castaneum* adults although four times less toxic to *S. zeamais* adults. 

In previous reports, (*R*)-carvone has been demonstrated to possess insecticidal activity against several species of insects and mites, e.g. Japanese termite (*R. speratus*) [12], sciarid fly (*L. ingenua*) [13], several stored product insects, rice weevil (*S. oryzae*), lesser grain borer (*R. dominica*), red flour beetle (*T. castaneum*) and flat grain beetle (*Cryptolestes pusillus*) [26,27,28,29], German cockroaches (*Blattella germanica*) [30] and the two-spotted spider mite, *T. urticae* [31]. D-Limonene has been commercialized for use as flea dips and shampoos for pets as well as sprays and aerosols [32]. It has been demonstrated to possess insecticidal activity against several stored-product insects such as the cowpea weevil (*C. maculates*), lesser grain borer (*R. dominica*), flat grain beetle (*C. pusillus*), rice weevil (*S. oryzae*), maize weevil (*S. zeamais*) and red flour beetle (*T. castaneum*) [28,29,33,34,35] as well as against the stored food mite *Tyrophagus putrescentiae* [36]. The two compounds were demonstrated to be a potent inhibitor of acetylcholinesterase (AChE) activity from larvae of several stored product insects [27,29].

Considering the currently used fumigants are synthetic insecticides, fumigant activity of the crude essential oil and the two isolated compounds are quite promising and they show potential for development as possible natural fumigants for the control of stored product insects. However, for the practical application of the essential oil/compounds as novel fumigants, further studies on the safety of the essential oil/compounds to humans and on development of formulations are necessary to improve the efficacy and stability and to reduce cost.

## 3. Experimental

### 3.1. General

^1^H and ^1^^3^C NMR spectra were recorded on Bruker Avance DRX 500 instruments using CDCl3 as solvent with TMS as internal standard. EIMS were determined on a ThermoQuest Trace 2000 mass spectrometer at 70 eV (probe). Silica gel (160-200 mesh) and pre-coated GF254 plates were purchased from Qingdao Marine Chemical Plant (Shandong Province, China). Fluon was purchased from ICI America Inc (USA). C_8_-C_24_ *n*-alkanes were purchased from Sigma-Aldrich (USA). All other unlabelled chemicals and reagents were of analytical grade.

### 3.2. Plant material

Fruits (1 kg) of *C. carvi* were purchased from Qinghai Chinese Medicinal Herbs Company, Qinghai Province, Xining 810000, China. The voucher specimen (BNU-CMH-Dushuahan-2009-08-25-006) was deposited at the Herbarium (BNU) of College of Life Sciences, Beijing Normal University. The fruits were air-dried for one week and ground to a powder for use.

### 3.3. Insects

The maize weevils (S. zeamais) and red flour beetles (T. castaneum) were obtained from laboratory cultures maintained in the dark in incubators at 29-30 °C and 70-80% r.h. The red flour beetles were reared on wheat flour mixed with yeast (10:1, w/w) while maize weevils were reared on whole wheat at 12-13% moisture content. Unsexed adult weevils/beetles used in all the experiments were about 2 weeks old.

### 3.4. Essential Oil Distillation

The ground powder of *C. carvi* fruits was subjected to hydrodistillation using a modified Clevenger-type apparatus for 6 h. Anhydrous sodium sulphate was used to remove water after extraction. Essential oils were stored in airtight containers in a refrigerator at 4 °C. The oil yields were 6.58% v/w.

### 3.5. Purification and Characterization of D-Limonene and (R)-Carvone

The crude essential oil was chromatographed on a silica gel column by gradient elution with *n*-hexane first, then with *n*-hexane-ethyl acetate, and last with acetone to obtain 25 fractions. Of these, fraction 6 and 9 were further separated by PTLC with petroleum ether-acetone (50:1, v/v) to afford two pure compounds, respectively. 

*D-Limonene* (**1**). Colorless oil. ^1^H-NMR (CDCl_3,_ 500 MHz) δ (ppm): 1.78 (3H, m, 10-CH_3_), 1.82 (3H, m, 7-CH_3_), 2.11 (1H, m, H-5), 2.39 (1H, m, H-3), 2.45 (1H, m, H-5), 2.57 (1H, s, H-3), 2.69 (1H, s, H-4), 4.76(1H, s, H-9), 4.81(1H, s, H-9), 7.65 (1H, s, H-6); ^13^C-NMR (CDCl_3_, 125 MHz) δ (ppm): 199.34 (C-2), 146.69 (C-8), 144.40 (C-6), 135.45 (C-1), 110.47 (C-9), 43.20 (C-3), 42.55 (C-4), 31.30 (C-5), 20.50 (C-10), 15.63 (C-7). Its identity was confirmed by the ESI-MS with the mass spectral fragmentation pattern [*m/z* (% abundance): 150(6.5), 108(31.9), 107(16.8), 106(11.4), 93(26.5), 82(100), 79(10.8), 54(47.6), 53(13.4), 41(18.5), 39(24.9), 27 (11.3)].


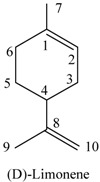


(*R*)-Carvone (**2**). Colorless oil. ^1^H-NMR (CDCl_3,_ 500 MHz) δ (ppm): 1.47 (1H, m, H-5), 1.65 (3H, s, 7-CH_3_), 1.73 (3H, s, 9-CH_3_), 1.79 (1H, m, H-5), 1.89 (1H, m, H-6), 1.97 (2H, m, H-3), 2.05 (1H, m, H-6), 2.08 (1H, m, H-4), 4.70 (2H, s, H-10), 5.40 (1H, s, H-2). ^13^C-NMR (CDCl_3_, 125 MHz) δ (ppm): 150.14 (C-8), 133.66 (C-1), 120.78 (C-2), 108.48 (C-10), 41.23 (C-4), 30.94 (C-6), 30.71 (C-3), 28.06 (C-5), 3.48 (C-7), 20.82 (C-9). Its identity was confirmed by the ESI-MS with the mass spectral fragmentation pattern [*m/z* (% abundance): 136 (22.6), 121 (19.5), 107 (17.1), 94 (22.6), 93 (59.1), 92 (18.8), 91 (12.7), 81 (10.9), 80 (11.1), 79 (22.8), 77 (11.7), 68 (100.0), 67 (44.7) 53 (17.1), 41 (19.3), 39 (15.6), 27 (10.3)].


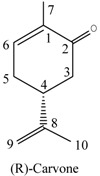


### 3.6. Gas Chromatography and Mass Spectrometry

Gas chromatographic analysis was performed on an Agilent 6890N instrument equipped with a flame ionization detector and HP-5MS (30m × 0.25mm × 0.25μm) capillary column, while the essential oil components were identified on an Agilent Technologies 5973N mass spectrometer. The GC settings were as follows: the initial oven temperature was held at 60 °C for 1 min and ramped at 10 °C min^−1^ to 180 °C for 1 min, and then ramped at 20 °C min^−1^ to 280 °C for 15 min. The injector temperature was maintained at 270 °C. The samples (1 μL) were injected neat, with a split ratio of 1:10. The carrier gas was helium at flow rate of 1.0 mL min^−^^1^. Spectra were scanned from 20 to 550 *m/z* at 2 scans s^−1^. Most constituents were identified by gas chromatography by comparison of their retention indices with those of the literature or with those of authentic compounds available in our laboratories. The retention indices were determined in relation to a homologous series of *n*-alkanes (C_8_–C_24_) under the same operating conditions. Further identification was made by comparison of their mass spectra with those stored in NIST 05 and Wiley 275 libraries or with mass spectra from literature [4,5,6,18]. Component relative percentages were calculated based on GC peak areas without using correction factors. 

### 3.7. Fumigant Toxicity

The fumigant activity of the essential oil and the pure compounds against *S. zeamais* and *T. castaneum* adults was tested as described by Liu and Ho [37]. Range-finding studies were run to determine the appropriate testing concentrations. A serial dilution of the essential oil/compound (0.05%-3.0% for pure compounds, 0.30-5.40% for oil, five concentrations) was prepared in *n*-hexane. A Whatman filter paper (diameter 2.0 cm) were each impregnated with 10 μL dilution, and then placed on the underside of the screw cap of a glass vial (diameter 2.5 cm, height 5.5 cm, volume 25 mL). The solvent was allowed to evaporate for 15 s before the cap was placed tightly on the glass vial, each of which contained 10 insects inside to form a sealed chamber. Fluon (ICI America Inc) was used inside glass vial to prevent insects from contacting the treated filter paper. Preliminary experiments demonstrated that 15 s was sufficient for the evaporation of solvents. *n*-Hexane was used as a control. Five replicates were carried out for all treatments and controls, and they were incubated for 24 h. The insects were then transferred to clean vials with some culture media and returned to the incubator and observed daily for determination of end-point mortality, which was reached after one week. The experiments were repeated in three times. The LC_50_ values were calculated by using Probit analysis [19]. 

### 3.8. Contact Toxicity

The contact toxicity of the essential oil/pure compounds against *S. zeamais* and *T. castaneum* adults was measured as described by Liu and Ho [37]. Range-finding studies were run to determine the appropriate testing concentrations. A serial dilution of the two essential oils (1.20%-5.0% for pure compounds, 1.50-5.20% for oil, five concentrations) was prepared in *n*-hexane. Aliquots of 0.5 μL of the dilutions were applied topically to the dorsal thorax of the insects. Controls were determined using *n*-hexane. Five replicates were carried out for all treatments and controls. Both treated and control insects were then transferred to glass vials (10 insects/vial) with culture media and kept in incubators. Mortality of insects was observed daily until end-point mortality was reached one week after treatment. The experiments were repeated in three times. The LD_50_ values were calculated by using Probit analysis [19].

## 4. Conclusions

Identified through mass screening, essential oil of *C. carvi* fruits and its major components were examined for insecticidal activity against the maize weevils and red flour beetles. The essential oil possessed strong fumigant toxicity against *S. zeamais* adults, although it was five times less toxic compared to the commercial fumigant MeBr. However, the essential oil exhibited the same fumigant toxicity to *T. castaneum* adults as MeBr. The two isolated compounds also exhibited strong fumigant toxicity against *S. zeamais* and *T. castaneum* adults. The essential oil and the two compounds also showed contact toxicity against the two species of grain storage insects. These findings, considered together, suggest that the essential oil and the two compounds show potential for development as natural fumigants for stored products.
